# Long-Term Consequences of Misdiagnosis of Parathyroid Adenomas in Pediatric Patients

**DOI:** 10.1155/crpe/2390925

**Published:** 2025-07-08

**Authors:** Dorota Roztoczyńska, Aleksander Konturek, Anna Wędrychowicz, Magdalena Ossowska, Alicja Kapusta, Anna Taczanowska-Niemczuk, Jerzy B. Starzyk

**Affiliations:** ^1^Department of Pediatric and Adolescent Endocrinology, Chair of Pediatrics, Institute of Pediatrics, Jagiellonian University Medical College, Kraków, Poland; ^2^Department of Endocrinological Surgery, 3rd Chair of Surgery, Jagiellonian University Medical College, Kraków, Poland; ^3^Department of Pediatric Surgery, Institute of Pediatrics, Jagiellonian University Medical College, Kraków, Poland

## Abstract

Primary hyperparathyroidism (PHPT) is rare in children but exhibits a more dynamic course than in adults, often leading to multiorgan complications if diagnosis is delayed. This article aims to highlight diagnostic challenges of parathyroid adenomas in children and discuss associated complications from delayed diagnosis. Three boys, aged 15.5, 10, and 16 years, were retrospectively analyzed for hypercalcemia. The diagnosis was based on biochemical and hormonal tests, as well as imaging studies (ultrasound, scintigraphy, and densitometry). The mean diagnosis delay was 20 months (9–35). All boys experienced appetite loss and bone symptoms. Patient 1 was initially misdiagnosed with slipped capital femoral epiphysis (SCFE) and underwent orthopedic surgery without recognition of severe hypercalcemia. Patient 2 was misdiagnosed with vasopressin deficiency following a tibia fracture. Patient 3's symptoms were attributed to stress. All patients had parathyroid adenomas, but Patients 2 and 3 had an ectopic thymus location. Following adenoma excision, Patients 2 and 3 developed hypocalcemia, the lasting consequences of which included nephrocalcinosis and low bone mass; Patient 3 also developed hypertension and depression. Conclusions: (1) any child presenting symptoms such as loss of appetite, abdominal pain, weight loss, depression, or bone abnormalities must urgently have serum calcium levels assessed to exclude PHPT. (2) Delayed diagnosis of PHPT in children is dangerous, as it leads to irreversible organ damage, including severe bone loss, nephrocalcinosis, and hypertension. (3) Comprehensive hormonal and genetic evaluation prior to surgery is essential, along with prompt correction of hypocalcemia to minimize complications and improve treatment outcomes. (4) After parathyroid adenoma removal, intensive calcium and vitamin D supplementation is required to prevent hungry bone syndrome and support proper bone recovery. (5) Due to the significant risk of disease recurrence, children with PHPT require long-term endocrine follow-up and thorough genetic testing, enabling early detection of relapse and timely intervention.

## 1. Introduction

Primary hyperparathyroidism (PHPT) is a rare endocrine disorder in the pediatric population, with an estimated incidence of 2–5 cases per 100,000 children, compared with 1 per 1000 in adults [1–6]. In contrast to adults, where PHPT is often asymptomatic, the disease in children typically follows a more aggressive course and presents with marked clinical symptoms. Up to 80% of affected children exhibit multiorgan complications by the time of diagnosis [6–10].

Delayed diagnosis can result in persistent complications, even after surgical treatment of the underlying cause. Nonspecific signs involving the skeletal, gastrointestinal, or urinary systems should prompt early evaluation of calcium levels and consideration of PHPT in the differential diagnosis.

This paper presents three pediatric cases of PHPT with atypical clinical presentation and initially incorrect diagnoses. The aim of this study is to highlight the diagnostic challenges posed by ectopic locations of parathyroid adenomas and the consequences of delayed diagnosis. We also emphasize the role of early genetic testing in ensuring safe surgical management and preventing disease recurrence.

## 2. Materials and Methods

The investigators conducted a retrospective clinical analysis of three boys hospitalized for hypercalcemia who were finally diagnosed with PHPT at the Department of Pediatric and Adolescent Endocrinology of the University Children's Hospital of Krakow in the years 2017–2022. The diagnosis of PHPT was made in these patients at the age of 15.5 years (Patient 1), 10 years (Patient 2), and 16 years and 10 months (Patient 3) based on the results of the following tests: total calcium (Ca total), phosphate, creatinine, alkaline phosphatase, urinary calcium excretion, serum parathyroid hormone (PTH), and vitamin D3 (25OH D_3_) values.

All the children underwent imaging tests, that is, ultrasound of the neck and abdominal cavity, SPECT/CT scans (99 mTc MIBI), neck and mediastinum scintigraphy, and spin densitometry using the DEXA (dual-energy x-ray absorptiometry) method. In our study, we always use adjusted Z-scores to assess bone densitometry results, recalculating the Z-score on the patient's bone age using the population reference standards provided by Hologic or Lunar, depending on the system used. In all patients, after appropriate diet, daily urine collections were performed to assess the excretion of catecholamines and their metabolites to exclude concomitant pheochromocytoma. Moreover, in all patients, serum levels of prolactin and chromogranin A were measured. In addition, genetic testing was conducted to exclude MEN 1 and MEN 2A syndromes.

Parental consent was obtained for all of the above tests and the tests themselves were performed in accordance with diagnostic procedures. Patients and their parents have agreed to the use of the test results and images for scientific purposes. Family history on PHPT was negative in all boys; the mother and grandmother of Patient 2 had a history of nephrolithiasis.

## 3. Results

**Figure 1:**
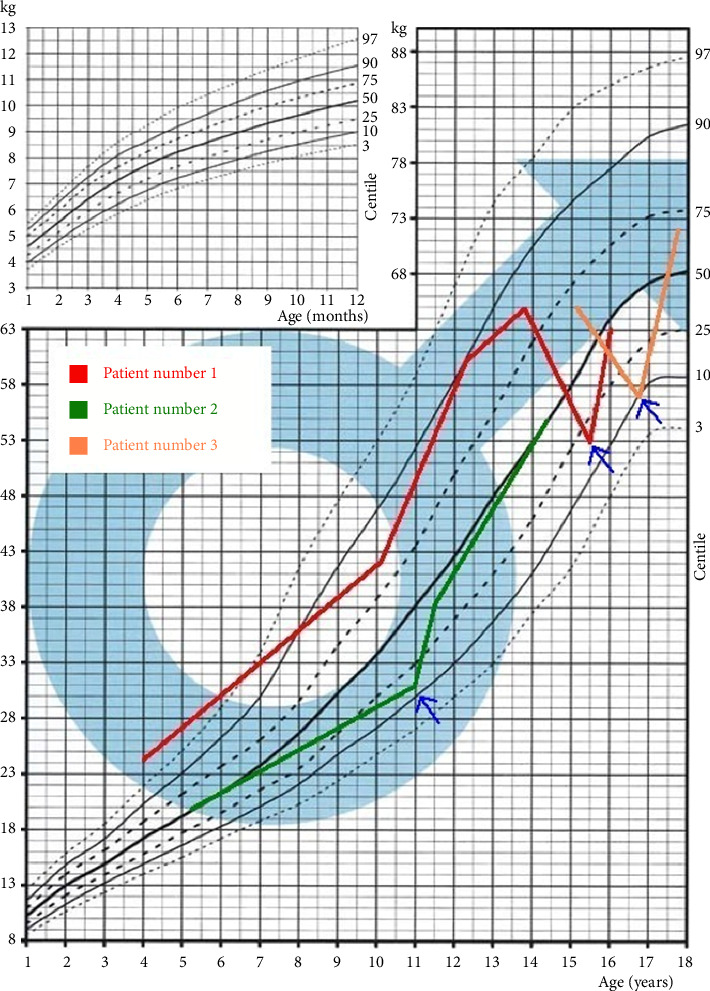
Graphs illustrating the body weight of Patients 1, 2, and 3 before and after surgical treatment for PHP. Arrows indicate the time of surgery. Body weight of Patients 1, 2, and 3 plotted against age (years) on a centile chart for boys aged 1–18 years.

The mean time from the onset of symptoms to diagnosis was 20 months (9–35 months) (Table 1). All patients presented with symptoms suggestive of gastrointestinal diseases, including lack of appetite, abdominal pain, weight loss, stomachache, and loss of body weight (Figure 1), as well as bone-related symptoms, such as bone pain, deformities, and fractures. When analyzing growth charts prior to diagnosis of PHPT, patients grew normally in accordance with the parental prognosis (midparental height (MPH)). All the boys had hypercalcemia, hypophosphatemia, hypercalciuria, and elevated PTH levels (Table 2). In all patients, malignant tumor-associated hypercalcemia was excluded as was pheochromocytoma. Genetic tests excluded the MEN 1 and MEN 2A syndromes. Patients 2 and 3 were diagnosed with nephrocalcinosis; Patient 3 was also diagnosed with hypertension and depression. The results of pre- and postoperative examinations are presented in Table 1.

### 3.1. Patient 1

**Figure 2:**
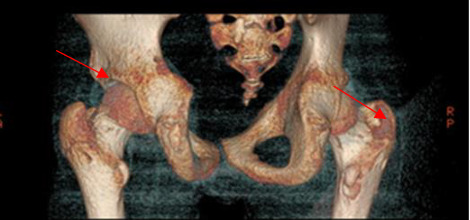
Bone structure loss in the left iliac bone above the hip joint space. Extensive bone structure loss in the greater trochanter of the right femur.

A 15.5-year-old boy had been experiencing bone pain, deformities impairing mobility, right-sided scoliosis, lack of appetite, constipation, and hypertension since October 2016 [8]. Between September 2016 and March 2017, he lost 10 kg (Figure 1). Over the course of 6 months, progressive valgus deformities developed in both legs, more pronounced in the left one. He was diagnosed with bilateral slipped capital femoral epiphysis (SCFE) at the Orthopedic Clinic. Bone scintigraphy and CT revealed generalized osteolysis, and a metabolic bone disease was suspected (Figure 2). However, the orthopedic team did not conduct diagnostic testing to determine the cause of the condition nor measure serum calcium levels, putting the patient at risk for a hypercalcemic crisis. In May 2017, bilateral subcapital osteotomy was performed, and the histopathological result indicated brown bone tumors. Only after the detection of brown tumors was an endocrinology consultation requested, and the patient was subsequently admitted to the Pediatric Endocrinology Department in Krakow.

**Figure 3:**
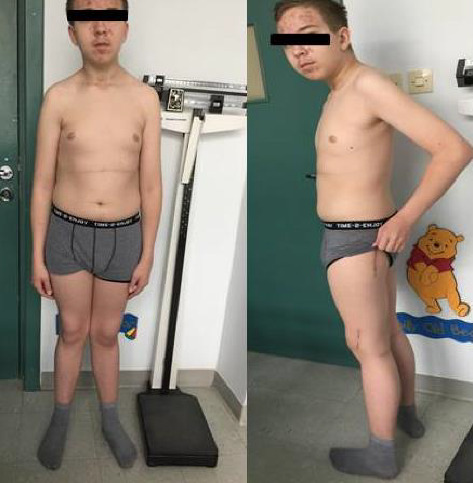
The 15-year-old patient with parathyroid adenoma after surgery due to bilateral slipped capital femoral epiphysis.

He was using crutches, had postsurgical scars on his legs, valgus knees, and right-sided thoracic scoliosis (Figure 3). His height was at the 25th percentile, with a 4% weight deficit (BMI: 0.5 SDS), and his puberty development was classified as stage 5 according to Tanner. His blood pressure was 140/80 mmHg. Serum PTH level prior to SPECT was markedly elevated at 310.1 pg/mL, further supporting the diagnosis of PHPT. Ultrasound and SPECT/CT with 99mTc-sestamibi revealed a 20-mm adenoma in the left lower parathyroid gland (Table 1). Severe hypercalcemia (Ca 4.07 mmol/L) and QTc shortening (0.34 s) were found, which required forced diuresis and loop diuretics without significant improvement. Normocalcemia was achieved after two doses of intravenous pamidronate (2 × 30 mg) (Table 2). Cinacalcet was not available in the department, so it could not be initiated before surgery.

The parathyroid adenoma was excised at the Department of Endocrinological Surgery, Krakow, 9 months after symptom onset. Postsurgery, the patient did not develop hypocalcemia but received calcium carbonate and vitamin D3. Six months later, his BMI improved to 1.2 SDS, and blood pressure normalized. After 2 years, BMD in the L2–L4 spine segment improved from −1.9 SD to −0.8 SD (Table 2). Now an adult, he undergoes genetic testing to determine the causes of PHPT.

### 3.2. Patient 2

A 10-year-old boy was admitted to the Department of Pediatric and Adolescent Endocrinology in February 2019 for hypercalcemia, polydipsia, and polyuria since January 2016, following a tibia fracture. He was initially diagnosed with diabetes and vasopressin deficiency due to polyuria, nocturnal enuresis, and epigastric pain. However, for 3 years, he was repeatedly diagnosed with these symptoms by his pediatrician, but calcium levels were never measured, which delayed the correct diagnosis. Physical examination showed height in the 75th percentile, a −24% weight deficiency (BMI: 1.4 SDS), and rotation of the right lower leg. Laboratory tests revealed hypercalcemia and elevated PTH (Table 1), while abdominal ultrasound showed nephrocalcinosis. The patient was diagnosed with PHPT, and cinacalcet was introduced, resulting in only a slight decrease in calcium levels (Table 2). Cinacalcet was initiated at a dose of 15 mg/day (0.5 mg/kg) and gradually increased to 30 mg/day (1 mg/kg). However, the patient poorly tolerated dose escalation, reporting persistent nausea and abdominal pain. Attempts to increase the dose above 30 mg/day led to vomiting. Despite treatment, serum calcium remained elevated (Ca: 3.18 mmol/L), and intravenous pamidronate was administered preoperatively, leading to effective normalization of serum calcium and enabling surgical intervention (Table 2).

Ultrasonography revealed a 4 × 3 mm hypoechogenic structure near the lower pole of the right thyroid lobe. Sestamibi scintigraphy (99mTc-MIBI) showed no increased tracer uptake, while CT scans identified a nodular lesion behind the right thyroid lobe, potentially a parathyroid gland. PET/CT indicated abnormal tracer accumulation in the same area. Intravenous pamidronate was given before surgery to achieve normocalcemia (Table 2).

Surgical treatment was performed at the University Children's Hospital. During the procedure, two nodular lesions adjacent to the right thyroid lobe were identified. The upper lesion was diagnosed as a parathyroid microadenoma, while the lower lesion consisted of ectopic thymic tissue with normal morphology, within which a parathyroid adenoma was present. Postoperatively, the patient experienced hypocalcemia, and calcium carbonate and active vitamin D3 were administered (Table 2).

The disease duration from PHPT onset to surgery was 3 years. Six months after surgery, the patient's weight increased to −0.28 SDS. Bone mineral density improved from a Z-score of −1.8–−1.1 SD over 3 years of supplementation (Table 2). Nephrocalcinosis persisted. Genetic diagnostics, including whole exome sequencing (WES), were performed, which excluded any genetic causes for the PHPT.

### 3.3. Patient 3

A 16-year-old boy was admitted to the Department of Pediatric Endocrinology in February 2022 due to hypercalcemia, hypertension, abdominal pain, nausea, and vomiting. In January, he sustained a fracture of the coronoid process of the right humerus after a fall. Earlier, his abdominal pain, nausea, and vomiting had been treated with Polprazole, providing temporary relief. Due to anemia, iron supplements were administered. Despite these treatments, abdominal pain recurred, leading to a referral to the Pediatric Gastroenterology Department, where hypercalcemia was finally diagnosed. Following this, the patient was referred to the Pediatric Endocrinology Department in Krakow. His height was at the 10th percentile, weight was appropriate for height, puberty development was at stage 5 according to Tanner, and his blood pressure was 164/85 mmHg. Laboratory tests confirmed hypercalcemia, hypophosphatemia, and elevated PTH levels (Table 2)

Neck ultrasound revealed a 20 × 16 × 9 mm vascularized nodular structure near the lower pole of the left thyroid lobe and an enlarged lymph node (Table 1). Abdominal ultrasound showed nephrocalcinosis. SPECT/CT with 99mTc-sestamibi confirmed focal tracer accumulation in the left lower parathyroid gland, measuring 14 × 11 mm, located below the thyroid and near the aortic arch Cinacalcet was administered but failed to normalize Ca and PTH levels. Intravenous pamidronate (2 × 30 mg) was used to achieve normocalcemia before surgery (Table 2). Surgery was performed at the Department of Endocrinological Surgery. Using a transverse incision, the surgeon identified and excised the left lower parathyroid gland along with the adjacent thymus lobe. Histopathological examination confirmed parathyroid adenoma without changes in the thymus. Postoperatively, hypocalcemia required increased calcium carbonate and active vitamin D supplementation (Tables 1 and 2).

The disease duration from symptom onset to surgery was 17 months. Six months postsurgery, BMI increased to 1.34 SDS, and after 1 year, BMD improved from a Z-score of −2.6–−1.5 in the L2–L4 spine segment (Table 2). The patient continues nephrological treatment for hypertension and nephrocalcinosis. In March 2023, his parents sought psychiatric consultation due to depression. The patient admitted to mood swings and depression for over 2 years, potentially linked to PHPT symptoms. Genetic testing for PHPT etiology is ongoing.

## 4. Discussion

Most publications emphasize the importance of early diagnosis of PHPT in children, as delayed recognition may lead to multiorgan complications resulting from chronic hypercalcemia [1–6]. According to the literature, the disease is diagnosed only after the onset of multiorgan complications in up to 80% of children with PHPT [6–9]. In the presented cases, Patient 1 did not develop any permanent organ complications following PHPT treatment, which may be related to the shortest disease duration (8 months), despite its rapid and severe course. Although cases of SCFE associated with PHPT have been sporadically reported in the literature, it is crucial to include serum calcium measurement in the differential diagnosis to prevent complications such as hypercalcemic crisis [5, 6, 10]. In Patient 2, a misdiagnosis of diabetes insipidus (vasopressin deficiency) was made, and serum calcium was not measured for 3 years. PHPT was diagnosed only after this period, delaying appropriate treatment and resulting in multiorgan complications. Similarly, in Patient 3, stress-related gastropathy was initially diagnosed, and hypercalcemia was detected incidentally during hospitalization. In both cases, diagnostic delays led to permanent complications despite treatment. In both boys, normalization of bone mineral density was not achieved, and they remain under nephrological care due to nephrocalcinosis. Additionally, Patient 3 requires treatment for arterial hypertension and has been referred for psychiatric consultation. The literature describes a correlation between PTH levels and aldosterone levels, where elevated PTH concentrations may induce increased aldosterone secretion, hypertension, and cardiovascular complications [11, 12]. Patient 3 had the highest PTH level (1380 pg/mL; normal: 10–60) and the highest blood pressure values, which did not normalize after surgery. His depressive symptoms may have coexisted with PHPT symptoms and resulted from chronic hypercalcemia [13]. The delay in diagnosing PHPT in children can be explained by the rarity of the disease during childhood and adolescence, which causes general practitioners, pediatricians, and orthopedic surgeons to seldom consider PHPT in the differential diagnosis. The most common symptoms of PHPT in children include fatigue, bone pain, pathological fractures, nephrolithiasis, polyuria, abdominal pain, nausea, vomiting, and weight loss [6, 8, 14, 15]. These symptoms should always prompt measurement of serum calcium levels, which is crucial for early diagnosis, effective treatment of PHPT, and reducing the risk of long-term complications. Early diagnosis of PHPT in children is particularly important due to the more severe and dynamic course of the disease compared to adults, in whom PHPT is often discovered incidentally. The literature emphasizes that children with PHPT typically have higher calcium and PTH levels than adults [6, 8, 14–17]. Tuli et al., which included a review of pediatric PHPT literature from 1982 to 2020, reported that single parathyroid adenomas were the most common cause of the disease, and the time to diagnosis—similar to our cases—exceeded 12 months. All children had total serum calcium levels > 3 mmol/L at diagnosis [6, 14–17]. Many publications highlight the frequent occurrence of ectopic parathyroid adenomas in children, including in the thymus, which poses a significant diagnostic challenge [18–21]. In an analysis of 86 children with PHPT treated between 1997 and 2017 (median age: 17 years), as many as 71% (*n* = 61) had a single adenoma confirmed histopathologically. In 25% of the cases (*n* = 22), the adenomas were ectopic—most commonly in the thymus (*n* = 13) but also in the tracheoesophageal groove, carotid sheath, and thyroid gland [18]. Scintigraphy with technetium-99m (99mTc) showed low sensitivity (10%) and high specificity (96%) for detecting ectopic adenomas; the positive predictive value (PPV) was 50% [19–24]. Other localization methods, such as neck ultrasonography and MIBI-SPECT, had a sensitivity of approximately 76% and 79%, respectively, with high PPVs (93% and 91%) [19]. The sensitivity of 99mTc-MIBI scintigraphy in children ranges from 68% to 86% [18–23]. In suspected mediastinal lesions, magnetic resonance imaging (MRI) is recommended, with a sensitivity of ∼88%, which exceeds that of MIBI scintigraphy (∼67%) [19–23]. It is recommended to use at least two, preferably three, imaging modalities to accurately localize the tumor in children [23]. PET with 18F-choline (18F-FCH) demonstrates higher diagnostic efficacy in adults, but its use in children is limited due to accessibility, cost, and the lack of clear guidelines [23, 24]. Genetic testing is an essential element in the diagnosis of PHPT in children. The disease may present in syndromic, isolated, familial, or sporadic forms. Approximately 50% of children with PHPT have detectable genetic mutations [25–28]. In neonates and young children, PHPT often results from inactivating mutations in the *CaSR* and *AP2S1* genes (NSHPT, FIHH1-3, and FIHP). In adolescents, mutations in *MENIN* (MEN1), *RET* (MEN2), *CDKN1B* (MEN4), and CDC73 (HPT-JT) predominate, usually leading to multiglandular parathyroid hyperplasia in syndromic forms of PHPT [25–28]. Genetic testing helps determine the risk of PHPT recurrence, and exclusion of MEN1 and MEN2A syndromes as well as thorough hormonal evaluation are essential for safe surgery. Therefore, these assessments were performed in all our patients. Genetic testing was extended to WES after surgical treatment. Effective preoperative management should include normalization of serum calcium levels to reduce perioperative risk. In the described cases, normocalcemia could not be achieved using cinacalcet. In Patient 2, vomiting occurred after increasing the dose from 0.5 to 1 mg/kg/day, and in Patient 3, high doses (2.3 mg/kg/day) were ineffective. Despite data indicating the efficacy and tolerability of cinacalcet in children [29], normocalcemia was only achieved preoperatively with intravenous bisphosphonates in our cases. Postoperative care in children with PHPT requires the use of high doses of vitamin D3 and calcium supplements to prevent hungry bone syndrome. In Patients 2 and 3, transient postoperative hypocalcemia occurred despite supplementation. In both boys, bone resorption related to PHPT lasted 35 and 17 months, respectively, leading to reduced bone mineral density on densitometry. The dosing and duration of supplementation should be individually tailored, and treatment efficacy should be monitored annually by densitometry, which should become a standard in the long-term endocrine care of these patients [30].

## 5. Conclusions

1. Every child presenting with symptoms such as poor appetite, abdominal pain, weight loss, depression, or skeletal abnormalities should promptly have their serum calcium level measured to rule out PHPT.
2. Delayed diagnosis of PHPT in children is dangerous, as it leads to irreversible organ damage, including severe bone loss, nephrocalcinosis, and hypertension.
3. Comprehensive hormonal and genetic evaluation prior to surgery is essential, along with prompt correction of hypocalcemia to minimize complications and improve treatment outcomes.
4. After parathyroid adenoma removal, intensive calcium and vitamin D supplementation is required to prevent hungry bone syndrome and support proper bone recovery.
5. Due to the significant risk of disease recurrence, children with PHPT require long-term endocrine follow-up and thorough genetic testing, enabling early detection of relapse and timely intervention.

## Figures and Tables

**Table 1:** Laboratory test results before and after surgical treatment and imaging findings.

No. of Patient	Patient 1	Patient 2	Patient 3
Patient's Age Findings	15 years and 6 months	10 years	16 years and 10 months
Preoperative PTH (N:10–60 pg/mL)	591.1	103.2	1380.0
Postoperative PTH (N:10–60 pg/mL)	41.5	12.3	129,1 (1 day after surgery) 42.2 (after 6 months)
Preoperative Ca (N:2.2–2.84 mmol/L)	4.07	3.53	3.48
Preoperative Ca DZM (N:0.03–0.1 mmol/kg/d)	—	0.24	0.16
Postoperative Ca (N:2.2–2.84) mmol/L	2.43	1.98 (first day after surgery) 2.53 (after 2 days)	2.08 (first day after surgery) 2.42 (after 2 days)
Preoperative P (N:0.95–1.75 mmol/L)	0.68	0.92	0.64
Postoperative P (N:0.95–1.75 mmol/L)	1.3	0.98	1.48
Preoperative 25 (OH) D3 (N: 30–60 ng/mL)	72	32	11.1
Postoperative 25 (OH) D3 (N:30–60 ng/mL)	25.1	41.7	34
Preoperative ALKP	—	185.5 U/L (N:120–488)	561 U/L (N:58–237)
Postoperative ALKP	136.3 U/L (N:58–237)	154 U/L (N:120–488)	60.5 (N:58–237)
USG of the neck	A hyperechogenic lesion 15 × 20.5 mm in size located in the inferior ridge of the left thyroid lobe	A small hypoechogenic structure 4 × 3 mm in size located paratracheally on the inferior ridge of the right thyroid lobe and directly communicated with the lobe	A nodular highly vascularized structure 20 × 16 × 9 mm in size situated at the inferior ridge of the left thyroid lobe (a longitudinal lymph node with intensified vascularity, 29 × 8 mm in size)
SPECT/CT (99 mTc MIBI) of the neck and mediastinum	Intensified marker uptake in the left inferior parathyroid gland.	No increased marker collection typical of adenomas seen within the parathyroid glands	Typical marker collection focus in the inferior left parathyroid gland, approximately 14 × 11 mm in size, located below the inferior ridge of the left thyroid lobe, between the brachiocephalic trunk and the left common carotid artery, approximately 10 mm above the aortic arch.
Results of additional imaging tests	TC99m-MRDP scintigraphy of the bones: increased uptake of markers in the metaphases, ribs, and flat bones. Bone CT–generalized osteolysis	CT of the neck and chest—a single nodular congestion located posteriorly to the right thyroid lobe that may correspond to the parathyroid gland PET/TK FGD: increased uptake of radiomarkers at the level of the postfracture fissure of the left inferior public ramus. In addition, there were areas of increased tracer accumulation at the level of Waldeyer's ring and posteriorly to the right lobe of the thyroid gland in the place described in the CT scan ring and left thymus level.	

**Table 2:** Type of pharmacotherapy used in patients before and after parathyroid adenoma resection and results of spinal densitometry of patients.

Patient	Preoperative pharmacotherapy	Postoperative pharmacotherapy	Preoperative densitometry Z-score L2–L4	Postoperative densitometry Z-score L2–L4
1	Pamidronate 2 × 30 mg, phosphate mix 3 × 5 mL/day, magnesium citrate 2 × 100 mg/day, cholecalciferol 4000 IU/day, magnesium citrate 2 × 100 mg/day, cholecalciferol 2000 IU/day	Cholecalciferol 6000 IU/day, alfacalcidol 1 μg/day, phosphate mix 3 × 5 mL/day, calcium carbonicum 2 × 1000 mg/day	−1.9 SD before surgery	−0.8 SD 2019 two years after surgery
2	Cinacalcet15 mg/day, pamidronate 2 × 30 mg	Cholecalciferol D 2000 IU/day, calcium carbonicum 3 × 2000 mg/day, alphacalcidol 3 × 1 μg/day	−1.8 SD before surgery	−1.3 SD 1 year after surgery −1.1 SD 2 years after surgery
3	Cinacalcet 3 × 30 mg/day, amlodipine besylate 7.5 mg/day, nebivolol hydrochloride 2.5 mg/day, magnesium citrate 2 × 100 mg/day, cholecalciferol 6000 IU/day, pamidronate 2 × 30 mg	Cholecalciferol 6000 IU/day, calcium carbonicum 3 × 1000 mg/day, alphacalcidol 1 × 1.0 μg/day, amlodipine besylate 7.5 mg/day, nebivolol hydrochloride 5 mg/day, magnesium citrate 2 × 100 mg/day	−2.6 SD before surgery	−1.5 SD 8 months after surgery

## Data Availability

The data used to support the findings of this study are available from the corresponding author upon reasonable request.
